# Novel hemodynamic structures in the human glomerulus

**DOI:** 10.1152/ajprenal.00566.2017

**Published:** 2018-06-20

**Authors:** Christopher R. Neal, Kenton P. Arkill, James S. Bell, Kai B. Betteridge, David O. Bates, C. Peter Winlove, Andrew H. J. Salmon, Steven J. Harper

**Affiliations:** ^1^Bristol Renal and School of Physiology, Pharmacology and Neuroscience, University of Bristol, Bristol, United Kingdom; ^2^Division of Cancer and Stem Cells, School of Medicine, University of Nottingham, Queen's Medical Centre, Nottingham, United Kingdom; ^3^Cardiff Centre for Vision Science, Cardiff University, Cardiff, United Kingdom; ^4^Nikon Imaging Centre, Guys Campus, Kings College London, London, United Kingdom; ^5^School of Physics, University of Exeter, Exeter, United Kingdom; ^6^Waitemata District Health Board, Auckland, New Zealand; ^7^Institute of Biomedical and Clinical Sciences, University of Exeter Medical School, Exeter, United Kingdom

**Keywords:** conduit vessels, glomerular microcirculation, hemodynamics, mesangial collagen, vascular chambers

## Abstract

To investigate human glomerular structure under conditions of physiological perfusion, we have analyzed fresh and perfusion-fixed normal human glomeruli at physiological hydrostatic and oncotic pressures using serial resin section reconstruction, confocal, multiphoton, and electron microscope imaging. Afferent and efferent arterioles (21.5 ± 1.2 µm and 15.9 ± 1.2 µm diameter), recognized from vascular origins, lead into previously undescribed wider regions (43.2 ± 2.8 µm and 38.4 ± 4.9 µm diameter) we have termed vascular chambers (VCs) embedded in the mesangium of the vascular pole. Afferent VC (AVC) volume was 1.6-fold greater than efferent VC (EVC) volume. From the AVC, long nonbranching high-capacity conduit vessels (*n* = 7) (Con; 15.9 ± 0.7 µm diameter) led to the glomerular edge, where branching was more frequent. Conduit vessels have fewer podocytes than filtration capillaries. VCs were confirmed in fixed and unfixed specimens with a layer of banded collagen identified in AVC walls by multiphoton and electron microscopy. Thirteen highly branched efferent first-order vessels (E1; 9.9 ± 0.4 µm diameter) converge on the EVC, draining into the efferent arteriole (15.9 ± 1.2 µm diameter). Banded collagen was scarce around EVCs. This previously undescribed branching topology does not conform to the branching of minimum energy expenditure (Murray’s law), suggesting that even distribution of pressure/flow to the filtration capillaries is more important than maintaining the minimum work required for blood flow. We propose that AVCs act as plenum manifolds possibly aided by vortical flow in distributing and balancing blood flow/pressure to conduit vessels supplying glomerular lobules. These major adaptations to glomerular capillary structure could regulate hemodynamic pressure and flow in human glomerular capillaries.

## INTRODUCTION

The control of glomerular blood flow is crucial for maintaining efficient ultrafiltration across the glomerular filtration barrier (GFB). Glomerular disease is characterized by molecular and physiological perturbations and altered glomerular hemodynamics (intraglomerular pressure and hyperperfusion); however, most of the models of glomerular hemodynamics in humans are based on experimental animals with small glomeruli. A few studies have attempted to reconstruct the human glomerular vascular network; a wax model of a human neonate glomerulus was reconstructed by Johnston in 1899 (21) and in 1956 plastic glomerular vessels were reconstructed from wax-molded outlines (6). These and later casting techniques render impressions of the glomerular surface capillaries with deeper vessels remaining largely hidden.

More recent computational methods have revealed nodes and branching in rat and human glomerular vasculature (33, 34, 47, 48, 53). The human reconstructions were performed on 5-µm sections and/or on immersion-fixed sources or only on small glomerular regions, and the few studies of the vascular pole of the human glomerulus have used biopsy or cadaver-recovered material (33, 56). To date, only one reconstructive study has been published using perfusion-fixation of a human transplant kidney but at elevated hydrostatic pressure (140 mmHg) where the authors chose a stereological approach for vessel analysis rather than reconstruction (4).

Glomerular capillaries operate at relatively high pressure in life, which in turn sets urinary driving pressure in the Bowman’s capsule and tubules producing tubular flow. For instance, the human glomerular capillary hydrostatic pressure of 60 to 65 mmHg at the afferent end (43) falls only 2–3 mmHg to the efferent end. Countering this filtration pressure is an afferent plasma colloid osmotic pressure of 25 mmHg rising to 32 mmHg at the efferent end (1). As a result of filtration, urinary space hydrostatic pressure is 20–25 mmHg (61) pressurizing the proximal convoluted tubule producing flow through to the collecting duct and the renal hilus. Thus, the function and structure of the whole nephron relies on the glomerular perfusion of an oncotically appropriate fluid at the correct hydrostatic pressure to raise the right physiological pressures and flows in the tubules. In biopsy/necropsy kidney specimens the absence of pressure during immersion fixation results in the collapse of both the glomeruli and tubules. Fixing at the correct physiological pressures (oncotic and hydrostatic) is therefore essential in investigating the true “functionally inflated” architecture of the glomerulus.

We have previously shown that three-dimensional ultrastructural reconstruction of animal and human glomeruli fixed under hydrostatic and oncotic physiological conditions allows the detailed analysis of the GFB and the identification of novel structural features such as the subpodocyte space (SPS) (39). One unexpected feature of light microscopic sections from these resin-embedded human glomeruli was the frequency of wide vessel regions at the vascular pole when compared with rodent vascular poles, implying different vascular structure. No mention of any such difference could be found in any recent study of human glomerular structure.

The hemodynamic requirements of rat and human glomeruli could shed light on any differing evolved morphologies. For instance, if glomerular volume is assumed to estimate perfused glomerular volume, this parameter does not scale in size with the increase in afferent arteriolar conductivity between rodents and humans. The human afferent arteriole has a conductivity 13-fold greater than that of the mouse (14,000 µm^4^ vs. 1,100 µm^4^) but supplies a 23-fold larger glomerular volume.^1^ Similarly, it is 3 times as conductive as that of the rat (4,600 µm^4^), while supplying a 5-fold larger glomerular volume. If human glomerular morphology was simply scaled up from a small rodent pattern, then afferent arterioles should be closer to 26 µm in diameter instead of 21 µm.

This study therefore aimed to investigate these novel wide vascular regions of human glomeruli. How big were these regions? What was the wall structure and dimensions and were there any other associated features? Did the region constitute a wider region at the base of the afferent arteriole or a region of a thin walled capillary? Could these structural differences be involved in compensating for a high glomerular volume relative to the vascular input in human glomeruli? To address such questions, human kidneys were perfuse fixed (at physiological hydrostatic and oncotic pressures) and processed in such a way to reduce any accompanying tissue volume changes. Glomerular vasculature was observed and reconstructions were made from fresh or fixed human kidney cortex using conventional light microscopy, confocal microscopy, multiphoton microscopy, and transmission electron microscopy.

## METHODS

### Fixation Techniques

Human kidney tissue was sourced (with full ethical approval and written informed consent of next of kin) from transplant kidneys (*n* = 9) unused for technical reasons (e.g., poor major vessel condition, damage at retrieval, tumor in the contralateral kidney). The transport solution perfused through the kidney was Soltran (potassium citrate 0.86% wt/vol, sodium citrate 0.82% wt/vol, mannitol 3.38% wt/vol, magnesium sulfate 1.0% wt/vol; Baxter Healthcare). Approximately 2–3 liters of the solution were perfused through the kidney (200 ml/min, 120–140 mmHg, 4°C) and then stored on ice. All other chemicals were sourced from Sigma-Aldrich.

Kidneys were transported in ice-cold flush media. Centimeter-diameter fresh cortical tissue was sampled from one pole for confocal and multiphoton microscopy and stored in chilled (4°C) HEPES-buffered Ringers solution. Smaller 1-mm-diameter tissue pieces were taken from the cut surface and fixed in 2.5% glutaraldehyde in HEPES buffer to serve as immersion-fixed samples for transmission electron microscopy. At 4–10°C, kidneys were debrided of excess fat preserving the hilar components (renal artery, vein and ureter), and the sampled polar area of the kidney was clamped off with a large locking forceps. The renal artery was cannulated and the renal vein was cleared of any debris to allow outflow of perfusion fluid.

To offset any hyperfiltration and hyperperfusion during fixation, normal hydrostatic and oncotic pressures were reestablished by perfusing with an oncotically balanced (25 mmHg oncotic pressure) flush solution (50 ml, 20°C). Colloid osmotic pressures were measured using a modified Hanson osmometer. The flush solution temperature was kept low to minimize autolytic/proteolytic activity. The hydrostatic pressure in the renal artery was set at 100 mmHg (similar to human mean arterial pressure). After the flush bolus, 400 ml of fixative was perfused through the kidney at the same pressures and temperature. Flush solution concentration was (mM) 132 NaCl, 4.6 KCl, 1.3 MgSO_4_, 2 CaCl_2_, 5 HEPES, 25 NaHCO_3_, 5.5 d-glucose, 6.5% (wt/vol) Ficoll 400. Fixative was the same as the flush solution but with 1.25% (wt/vol) glutaraldehyde. The glycocalyx stain 0.5% lanthanum nitrate and 0.5% dysprosium chloride was incorporated into the solutions in two kidneys.

One-millimeter-diameter samples of perfusion-fixed kidney were taken from a medial subcapsular position and together with subcapsular immersion-fixed samples were postfixed in osmium tetroxide, dehydrated with ethanol, and processed into Araldite resin using standard procedures.

To promote consistency in structural comparisons, measurement and observations were limited on the glomeruli of the outer (subcapsular) cortex of kidneys in a medial location halfway between the poles (unless otherwise stated).

### Reconstruction of Vascular Poles From Perfusion-Fixed Kidneys

Seven areas of resin-embedded kidneys (*n* = 4) which contained a high density of glomeruli were identified in Toluidine Blue stained sections. These areas were serially sectioned on a Reichert Ultracut microtome at 1 µm thickness (2,095 sections approximately 300 sections per area). From these serial section runs, three or four fully sectioned glomeruli from each kidney were selected that clearly showed a vascular pole. The afferent arterioles of each of the 14 glomeruli were identified by tracing to a larger artery and/or the efferent arteriole traced to a peritubular position.

Digital micrographs (1,834) of serial sections of glomeruli (*n* = 14) were made using a ×40 objective on a Nikon E400 microscope. Digital images were repositioned, aligned, calibrated, and measured using ImageJ software (NIH open source ImageJ 1.46r and 1.47o; NIH, Bethesda, MD) and compiled into image stacks. Topological maps were made of the route and diameter of the blood vessels coursing through the afferent and efferent parts of the vascular pole.

### Resin Section Thickness Calibration and Glomerular Diameter

Measurement and reconstruction in the sectioning direction is reliant on the precision of the ultramicrotome mechanism controlling section thickness. To test the accuracy of the ultramicrotome, glomeruli were assumed to be spherical and of similar diameter in all directions. Glomerular diameter was measured in the sectioning direction (*z*) as well as in the section plane (*x*,*y*). An ellipse was fitted over the largest glomerular profile of a section image (*x*,*y*), maximum and minimum diameters were measured from this, and results were pooled (194.4 ± 5.1 µm *n* = 28). In the image stacks of a glomerulus the first and last sections to contain the edge of glomerular blood vessels were found and the number of intervening sections were counted (202.4 ± 5.0 *n* = 14). Assuming 1 µm section thickness, there was no significant difference between the estimates of glomerular diameter from either method (*P* = 0.325, *t*-test) and no correction was needed for section thickness or measurements of length in the sectioning direction (*z*).

The glomerular diameters (2*r*_x_ 2*r*_y_ 2*r*_z_) measured during the calibration of section thickness were used to calculate glomerular volume (V_G_ = 1.33 π *r*_x_ *r*_y_ *r*_z_).

### Glomerular and Vascular Orientation in Resin Section Reconstruction

Vascular pole recognition was most easily achieved in 1 µm serial resin sections where the section plane was paraxial with the vascular pole–urinary pole axis of the glomerulus; as a result, reconstructed glomeruli were sectioned close to a paraxial plane. The true diameters of any vessel profile were measured by searching the sequential images for the appropriate vessel section and measuring vessel width (*x*,*y*). Section depth diameter was taken from the limits of vessel walls in the sectioning direction (*z*). Vessel lengths (between branch points for example) through the image stack were measured on section if possible or by triangulating through the stack using sectioning depth and horizontal “on section” distance.

The three diameters of vascular chambers (VCs; *x*, *y*, and *z*) used to calculate the means in Tables 1 and 2 were further used to calculate afferent and efferent vascular chamber volume (V_AVC_ = 1.33 π *r*′_AVC_ *r*′′_AVC_ *r*′′′_AVC_; V_EVC_ = 1.33 π *r*′_EVC_ *r*′′_EVC_ *r*′′′_EVC_).

**Table 1.** Afferent vascular diameters

	Arteriole (AA)	Vascular Chamber (AVC)			First-Order (Conduit) Vessels	
	Diameter (2 *r*), µm	Min Diam (2 *r*′), µm	Secn Depth Diam (2 *r*′′), µm	Max Diam (2 *r*′′′), µm	Diameter (2 *r*), µm	*n*
Mean	21.5	32.1	49.4	48.0	15.9	6.6
SE	1.2	1.5	3.4	3.6	0.7	0.6

Diameters of afferent vessels from resin-embedded glomeruli (14) from 4 human kidneys. In all cases the afferent arteriole widened to form an ellipsoidal chamber with between 2 and 11 high-capacity conduit vessels emerging and conveying fluid away to the filtration capillaries. In the 14 glomeruli analyzed, 2 extra wide conduit vessels (19–24 µm) were found. Vascular chamber dimensions: Min Diam, minimum diameter measured in the section plane avoiding oblique vessel sections; Max Diam, maximum diameter measured in the section plane avoiding oblique vessel sections; Secn Depth Diam, diameter measured in the sectioning direction. AA, afferent arteriole; AVC, afferent vascular chamber; SE, standard error of the mean.

**Table 2.** Efferent vascular diameters

	Arteriole (EA)	Vascular Chamber (EVC)			First-Order Vessels (E1)	
	Diameter (2 *r*), µm	Min Diam (2 *r*′), µm	Secn Depth Diam (2 *r*′′), µm	Max Diam (2 *r*′′′), µm	Diameter (2 *r*), µm	*n*
Mean	15.9	26.2	45.9	43.1	9.9	12.6
SE	1.2	1.4	9.1	4.3	0.4	1.4

Diameters of efferent vessels from resin-embedded glomeruli (14) from 4 human kidneys. In all cases the efferent arteriole widened to form an ellipsoidal chamber, blood from the filtration capillaries converged into 3 to 22 narrow efferent first-order vessels (E1) these vessels drained into the efferent vascular chamber and thence the efferent arteriole. In the 14 glomeruli analyzed, 1 extra wide E1 vessel (20–27 µm) was found but the branching was frequent as in other E1 vessels. Vascular chamber dimensions: Min Diam, minimum diameter measured in the section plane avoiding oblique vessel sections; Max Diam, maximum diameter measured in the section plane avoiding oblique vessel sections; Secn Depth Diam, diameter measured in the sectioning direction. EA, efferent arteriole; EVC, efferent vascular chamber; SE, standard error of the mean.

Bends between arterioles and VCs were assessed in resin section image stacks of 10 glomeruli by assessing the afferent and efferent arteriole axis vector and measuring the change in angle into the VC axis vector (Fig. 4*A*). This included measurements on section and in the sectioning direction and triangulation in vessels moving at angles to the section plane.

### Afferent First-Order (Conduit) Vessel Ballooning in Resin Sections

Any ballooning or hyperinflation of first-order afferent (conduit) vessels was estimated initially by comparing conduit diameters in areas of potentially high transmural pressure gradient (conduit vessels with large areas of GFB, 0–60% mesangial cover) with conduit diameters in areas of potentially low transmural pressure gradient (conduit vessels with 80–100% mesangial cover). These data were further dissected in each conduit vessel by subdividing the initial 0–60% mesangial cover group into four groups and using the 80–100% mesangial cover group as a baseline to calculate the fold change in diameter.

### Podocyte Cell Body Coverage of Conduit Vessels

Podocyte cell body (PCB) coverage on the urinary side of conduit vessels was estimated by measuring length of GFB in a vessel image covered by a visible podocyte cytoplasmic region and the accompanying areas where no cell body was apparent. This was compared with similar measurements from filtration capillaries.

### VC Recognition in Single Resin Sections

To test whether evidence of VCs could be seen in single sections of glomeruli (being the more common way of looking at human biopsy glomeruli), the occurrence of widened vasculature at the vascular poles was assessed in single sections of renal cortex. In an additional 13 resin-embedded human kidneys, immersion- and perfusion-fixed single cortical sections (1 µm thick) were stained with Toluidine Blue. Glomerular sections showing vascular poles were assessed for the frequency of vascular widening around the poles. Width was assessed by placing an ellipse around widened vascular profiles and taking the minimum diameter to eliminate oblique vascular diameter measurements.

### Confocal and Multiphoton Light Microscopy on Fresh Kidney Slices

Aqueous fresh and fixed kidney was observed using confocal and multiphoton microscope techniques. A Nikon confocal microscope (Nikon Eclipse Ti) was set to image fixation-induced autofluorescence (FIA). Millimeter and submillimeter thick fixed renal cortical slices were washed in HEPES Ringer solution, and the autofluorescent signal (FIA) at 488 nm wavelength was used to image and obtain *z*-stacks from glomerular vascular poles of up to 100 µm depth from the cut surface.

Using a multiphoton microscope, two fresh and two fixed unstained slices of renal cortex, were imaged as previously described (2). Two imaging modes were applied, fibrous collagen was visualized using second harmonic generation (SHG), and elastin was visualized from its intrinsic two-photon fluorescence (TPF) along with any background fluorescence. TPF and SHG images were obtained using a modified confocal microscope (FluoView IX71 and F300, Olympus). Signal was produced using the 800 nm output of a mode-locked Ti:sapphire laser (Mira 900-D, Coherent) pumped by a 532 nm solid state laser (Verdi V10, Coherent). The pulsed laser had a pulse width of 100 fs and a repetition rate of 76 MHz. The light was focused on to the sample using a ×60 1.2 numerical aperture water immersion objective (UPlanS Apo; Olympus). Signal was collected in the epi-direction using the objective lens and was separated from the laser fundamental using a long pass dichroic mirror (670dcxr; Chroma Technologies). The signal was then passed through two filters (for TPF: CG-BG-39 and F70-500-3-PFU; and for SHG: CG-BG-39 and F10-400-5-QBL; CVI Laser) before being focused on a photomultiplier tube (R3896, Hamamatsu). Each 1,024 × 1,024 pixel image took 29 s to acquire, meaning that a stack of 100 images, each separated in the *z*-direction by 1 μm, took ~50 min to complete.

### Electron Microscopy

From 1 µm resin sections of renal cortex showing identifiable VCs, further sections were cut at 70–100 nm thickness and stained with 10% phosphotungstic acid (10 min). Sections were viewed and digital images were taken on a Tecnai T12 (FEI).

### Calculation of Vascular Resistance

The resistance to flow along the terminal part of the arterioles will change as blood enters AVCs and conduits and exits EVCs. Assessing such resistances may give a better understanding of how blood flow will be affected by VCs and conduits; a correlate of total conduit resistance per unit length (*R*′_Con_) was derived from the Poiseuille equation (see appendix a)

(1) $$R\prime_{\text{Con}}=\frac{1}{r_{\text{Con}}{}^{4}\cdot n_{\text{Con}}}$$

where *r*_Con_ is the mean conduit vessel radius and *n*_Con_ is the number of conduit vessels merging from an AVC. *R*′_Con_ provides a value that scales proportionately with total vascular resistance per unit length. Similarly, a correlate of first-order efferent (E1) resistance per unit length (*R*′_E1_) was estimated from 1/*r*_E1_^4^ *n*_E1_ and arteriole resistance per unit length (*R*′_AA_, *R*′_EA_) was calculated from 1/*r*_AA_^4^ and 1/*r*_EA_^4^.

### Statistics

Data are represented throughout as either mean ± SE or as median (interquartile range). Excel was used for collating data and initial statistics, and Prism software (GraphPad Software) was used for statistical analysis generating histograms, correlations, and parametric and nonparametric tests.

## RESULTS

### Glomerular Structure From Resin Serial Section Image Stacks

#### Glomerular arterioles.

**Fig. 1.**
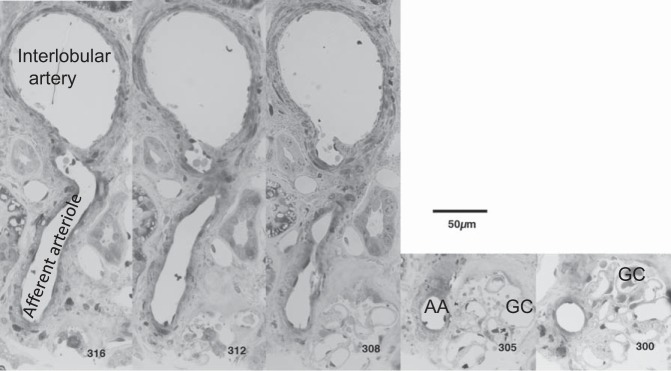
Afferent arteriole and glomerulus connectivity. Selected light micrographs from a 1 µm serial section stack to show the connectivity of an afferent arteriole (25 µm diameter) with a small artery (110 µm diameter interlobular or feed artery). Identifying the root/route of the vessels entering the glomerulus allows identification of afferent and efferent arterioles. Note that the afferent arteriole goes through a right angle as it enters the glomerulus. AA, afferent arteriole; GC, glomerular capillary. Serial section number at *bottom right*.

Assigning afferent and efferent labels to arterioles was accomplished by tracing the origin of these vessels in the serial section image stacks. Branches of cortical radial or interlobular arteries (38, 58) were traced to the afferent arterioles (Fig. 1) and efferent vessels showed a characteristic peritubular branching course on emerging from glomeruli.

**Fig. 2.**
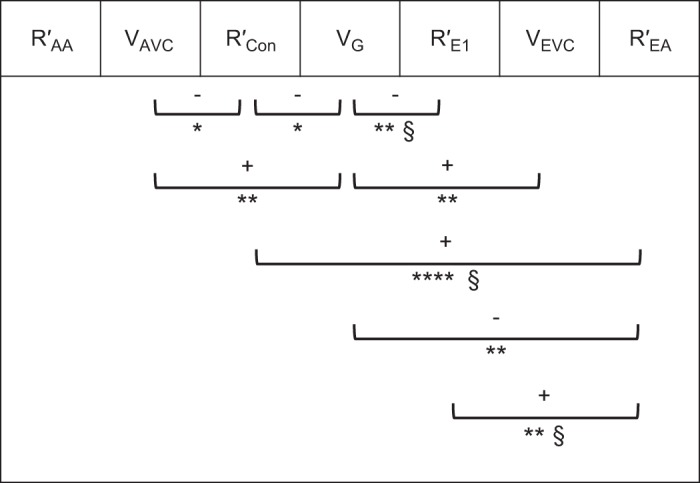
Vascular resistance and capacity relationships. Significant correlationships (8 out of 21) between 7 variables measured in human glomerular initial vasculature. Correlates of vascular resistance for afferent arterioles (R′_AA_), conduit vessels (R′_Con_), 1st-order efferent vessels (R′_E1_), and efferent arterioles (R′_EA_) were compared with each other and with afferent vascular chamber (AVC) volume (V_AVC_), glomerular volume (V_G_), and efferent vascular chamber (EVC) volume (V_EVC_). +, Positive correlation; –, negative correlation. *P < 0.05; **P ≤ 0.01; ****P ≤ 0.0001; §higher significance with outlier removed.

Afferent and efferent arteriole wall thicknesses were significantly different (6.6 ± 0.3 µm, 3.0 ± 0.1 µm, respectively, *n* = 7; *P* < 0.0001, paired *t*-test) as were afferent and efferent luminal diameter (23.2 ± 1.8 µm, 17.6 ± 2.0 µm, respectively, *n* = 7; *P* = 0.02, paired *t*-test), wall thickness being a better predictor of arteriole type than luminal diameter. No correlation was found between the afferent (*R*′_AA_) and efferent (*R*′_EA_) arteriole resistance measure (Fig. 2; *R*^2^ = 0.033, *P* = 0.53).

The efferent picture was confused by multiple efferent arterioles in 4 out of 14 glomeruli. Major efferent arterioles are shown in Table 2; the extra 1 to 3 minor efferents were in series or parallel with EVC and were 4.6–8 µm diameter with one 11.5 µm in series with an efferent VC. No extra afferent arterioles were seen.

#### Reconstruction of VCs and first-order vessels.

**Fig. 3.**
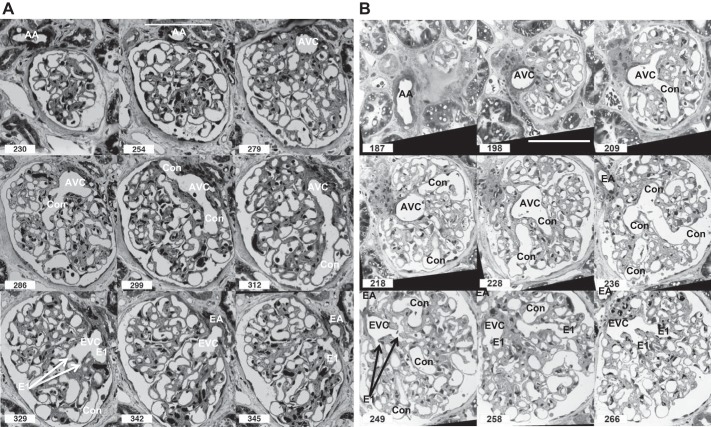
*A* and *B*: serial resin sections through a glomerulus. Selected light micrographs from 2 complete 1 µm serial section series to show the route that blood takes from an afferent arteriole (AA) into an afferent vascular chamber (AVC) leading into conduit vessels (Con) of high capacity and few branches. At the other end of the microcirculation, many branching efferent 1st-order vessels (E1) drain into a smaller efferent vascular chamber (EVC) leading to an efferent arteriole (EA). Serial section numbers at *bottom left*. Scale bar, 100 µm in micrograph of section 254 or 198 (see Supplemental Videos S2A and S2B for glomerular image stacks of *A* and *B*, respectively, and Supplemental Videos S2C and S2D for a reconstruction of afferent and efferent parts of *B*).

All 14 glomeruli (4 kidneys) analyzed from image stacks of 1 µm resin serial sections showed afferent and efferent widening of the arterioles, resulting in vascular chambers (VCs) embedded in the mesangium of the vascular pole (Fig. 3, see Supplemental Videos S2A and S2B for full image stacks; Supplemental Material for this article is available online at the Journal website). Some afferent VCs (AVCs) protruded into a hilar or juxta-glomerular position (sections 198 and 209, Fig. 3*B*).

**Fig. 4.**
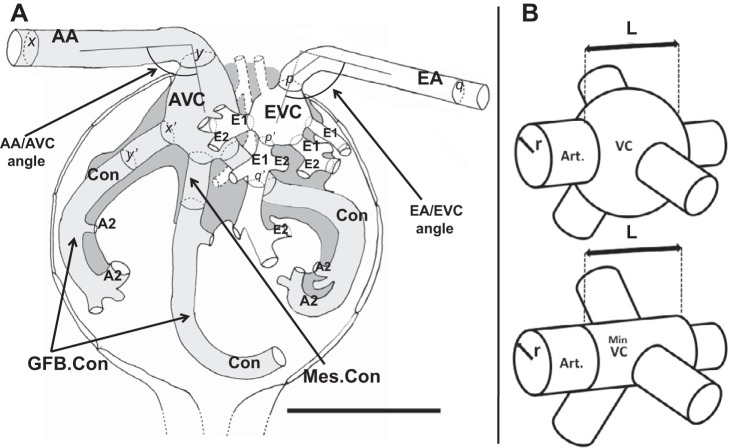
Scale diagram of glomerular vasculature; the smallest vascular chambers (VCs). *A*: scale diagram of the afferent (light gray) and efferent (white) ends of the glomerular vasculature. Diagram shows size and branch relationships between arterioles, VCs, and 1st-order vessels (mesangium close to vascular pole, dark gray) (diameters from Tables 1 and 2). To illustrate VC volume in relation to attached vessels, the length of attached vessels accommodating VC volume is shown; afferent VC volume (V_AVC_) would distribute along 112 µm length (delimited by hoops *x, y*) of afferent arteriole (AA) or distribute along 31 µm length (delimited by hoops *x*′*, y*′) of 7 conduit vessels (Con; 3 of 7 shown). The efferent VC volume (E_AVC_) would fill 138 µm length of efferent arteriole (EA; hoops *p, q*) or 28 µm length of 13 1st-order vessels (E1; hoops *p*′*, q*′, 4 of 13 shown). Scale bar, 100 µm. A2 and E2, 2nd-order vessel examples; Mes.Con, conduit vessel embedded in mesangium; GFB.Con, conduit vessel with glomerular filtration barrier (GFB) surface and minor mesangial attachment. *B*: minimal vascular chambers. *Top*: VCs shown as in our reconstructions, but both V_AVC_ and V_EVC_ decrease as glomerular volume (V_G_) decreases (Fig. 5, *C* and *D*). VC shrinkage in the radial direction would reduce the diameter and VC volume until it was a continuation of the attached arteriole.

**Fig. 5.**
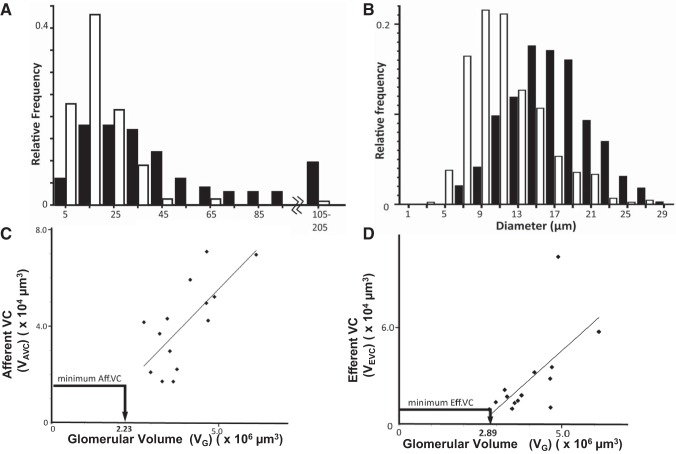
Conduit branching and diameter; vascular chamber (VC) volume scales with glomerular volume (V_G_). *A*: histogram of branch separation between 2nd-order branches (A2 or E2) emerging from 1st-order vessels (Con or E1). Branch intervals were assessed in 9 glomeruli. Conduit vessels (Con, closed bars) are longer and less branched than 1st-order efferent vessels (E1, open bars) (Mann-Whitney *U*-test medians (32.8, 15 µm), *P* < 0.0001). *B*: histogram of 1st-order vessel diameter coming off vascular chambers. Conduit vessels (closed bars) are significantly wider than efferent 1st-order vessels (open bars). Efferent distribution is skewed towards lower values [15.3 (12.8–18.9) vs. 9.0 (7.0–11.1); median (interquartile range); Mann-Whitney *U*-test, *P* < 0.0001]. *C* and *D*: afferent VC volume (V_AVC_; *C*) and efferent VC volume (V_EVC_; *D*) scale with glomerular volume to a highly significant level (*R*^2^ = 0.517 *P* = 0.004; *R*^2^ = 0.419 *P* = 0.012, respectively). A minimum possible V_AVC_ and V_EVC_ (see Fig. 4*B*) is also plotted to show V_G_ where VCs are a continuation of the attached arteriole (i.e., no VC widening).

Vascular width and connectivity are illustrated in a scale diagram in Fig. 4*A*, (measurements from Tables 1 and 2). To summarize, the 21 µm diameter afferent arteriole (AA) leads into an ellipsoidal afferent vascular chamber (AVC; 49×48×32 µm) which branches into on average 7 first-order afferent vessels of 16 µm diameter we have termed conduit vessels (Con; Fig. 3, *A* and *B*, Fig. 4*A*, and Table 1). These vessels had secondary vessels (A2) emerging at spacings of 32.8 µm (median), with 41% of branches intervals between A2 greater than 40 µm with a quarter of these above 100 µm (Fig. 5*A*). Conduit branches into A2 were more frequent distal to the AVC at the glomerular edge (Fig. 3*B* and Supplemental Videos S2A and S2B). Conduit vessels coursed through mesangium and then either through the center of the glomerulus or peripherally over the glomerular surface before branching into capillary networks (Fig. 3, *A* and *B*) [Supplementary Videos S2C and S2D (Fig. 3*B* as a reconstruction)].

At the efferent end of the filtration capillary network, first-order efferent vessels (E1) were more numerous (13 vs. 7) and narrower than conduits (10 µm vs. 16 µm diameter; Fig. 5*B* and Tables 1 and 2). Secondary efferents (E2) merged at 15 µm intervals into 13 first-order vessels (E1) (Fig. 3, *A* and *B*, and Fig. 4). Only 4% of E2 branch intervals on E1 vessels were above 40 µm - (Fig. 5*A*). E1s converged into an efferent vascular chamber (EVC; 46×43×26 µm) in turn disgorging into a 16 µm diameter efferent arteriole (EA; Fig. 3, *A* and *B*, Fig. 4*A*, and Table 2).

In 10 of the 14 glomeruli where the orientation of afferent and efferent arterioles on entry into the VCs could be easily assessed, the AA bent 60° off its straight track into the AVC (AA/AVC angle = 120 ± 6°); similarly, the EA bent 71° off track into EVC (EA/EVC angle = 109 ± 7°; Fig. 4*A*).

#### VC and glomerular size.

AVC volume (V_AVC_ = 41 ± 5 × 10^3^ µm^3^) was 1.6-fold greater than EVC volume (V_EVC_ = 28 ± 7 × 10^3^ µm^3^), with no correlation between them (Fig. 2; *R*^2^ = 0.164, *P* = 0.152). V_AVC_ varied over a greater size range (15–70 × 10^3^ µm^3^) with V_EVC_ more conserved (12 out of 14 between 10 and 40 × 10^3^ µm^3^). Both V_AVC_ and V_EVC_ correlated significantly with V_G_ (Fig. 5, *C* and *D*, and Table 3), V_G_ being 100-fold larger than V_AVC_ and 150-fold larger than V_EVC_. This implies a relationship between the magnitude of the perfused volume and the input and output manifolds.

**Table 3.** Vascular diameters and wall thicknesses - all experiments

		AA	AA	AVC	AVC Coll	Conduit	E1	EVC	EA	EA
	*n*	Diameter, µm	Wall Thickness, µm	Diameter, µm	Wall Thickness, µm	Diameter, µm	Diameter, µm	Diameter, µm	Diameter, µm	Wall Thickness, µm
Fixed Resin Reconstruction	14G, 4K	21.5 ± 1.2	6.6 ± 0.3	43.2 ± 2.8	*	15.9 ± 0.7	9.9 ± 0.4	38.4 ± 4.9	15.9 ± 1.2	3.0 ± 0.1
Fixed Aq. Confocal	4G*, 1K	28.4 ± 1.9	6.3 ± 0.8	35.8 ± 3.5	*	16.0 ± 1.2	8.2	24.2	12.8	*
Fixed Aq. Multiphoton	3G*, 1K	*	*	50.2 ± 3.7	4.2 ± 0.8	12.8 ± 1.6	6.9	28.1	7.4	*
Fresh Aq. Multiphoton	3G*, 2K	13.8	3.0	35.8 ± 4.1	4.1 ± 1.9	14.4 ± 0.9	*	*	*	*
Fresh Aq. Multiphoton	Isolated 1 G*, 1K	23.0	*	54.2	2.5	27.4	*	*	*	*

Comparison of afferent arteriole (AA), afferent vascular chamber (AVC), Conduit, efferent first-order vessels (E1), efferent vascular chamber (EVC), and efferent arteriole (EA) measurements from resin section reconstruction with the same features in fixed and fresh glomeruli in buffered aqueous media (Aq.) reconstructed from confocal and multiphoton microscope *z*-stacks (second harmonic generation, SHG, and two-photon fluorescence, TPF). EVC and AVC values have been averaged together for all 3 axes. AVC collagen sheath (AVC Coll) enshrouded AVC and some parts of conduit vessels but scant evidence in EVC or E1 (multiphoton microscopy only). G and K indicate numbers of glomeruli and kidneys used.

* Not all quantities were observable and measureable.

If the glomerular and VC volume (Fig. 5, *C* and *D*) correlation is extrapolated back from larger glomeruli then a minimal VC volume can be reached where the volume describes a mere continuation of the attached arteriole (Fig. 4*B*). Accordingly, a cylindrical minimum VC volume was calculated using average VC length (*L*) and arteriole radius (*r*); a minimum AVC volume of 1.57 × 10^4^ µm^3^ would occur at a V_G_ of 2.2 × 10^6^ µm^3^ (Fig. 5*C*). Similarly, a minimum EVC volume of 0.75 × 10^4^ µm^3^ would occur at a V_G_ of 2.9 × 10^6^ µm^3^ (Fig. 5*D*). Translating V_G_ into glomerular diameter, VCs would be minimal (a continuation of the arteriole) in human glomeruli below 160–180 µm diameter (i.e., V_G_ = 2–3 × 10^6^ µm^3^ - arrows on VG axes in Fig. 5, *C* and *D*).

#### Conduit podocytes.

**Fig. 6.**
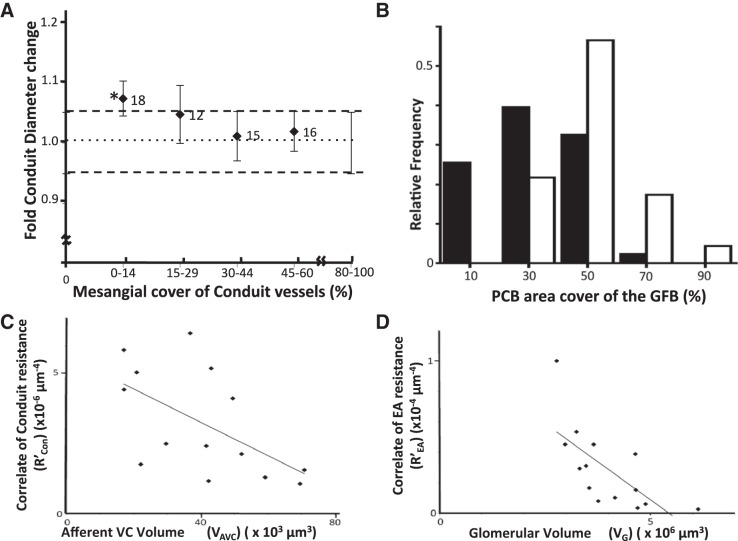
Conduit diameter changes with mesangium; conduit podocyte attachment; resistance vs. capacity examples. *A*: conduit diameter changes relative to mesangial cover. Conduit vessel diameters adjacent to the afferent vascular chamber (VC) with mesangial cover of 80–100% [glomerular filtration barrier (GFB) coverage 0–20%] were compared with diameters of low mesangial covered (distal) regions of the same vessel. The fold change in diameter shows a significant diameter increase of 7.4% (*) when mesangial cover is minimal (0–14%, i.e., GFB 86–100%). *P* = 0.04, paired *t*-tests and Wilcoxon matched pair test. *B*: histogram of podocyte cell body (PCB) area coverage of the filtration barrier of conduit vessels (closed bars) and small filtration capillaries (open bars). Conduits have significantly less PCB coverage of the GFB than filtration capillaries (*P* < 0.0001, *t*-test). *C*: conduit resistance vs. afferent VC volume (V_AVC_). A significant negative correlation exists between a correlate of conduit resistance (*R′*_Con_) and V_AVC_ (*R*^2^ = 0.327, *P* = 0.033). *D*: efferent arteriole resistance per unit length (*R′*_EA_) reduces in line with increasing glomerular volume (V_G_; *R*^2^ = 0.47, *P* = 0.007).

In resin section image stacks spanning a conduit vessel, we noted a significant lack of coverage of podocyte cell bodies (PCB) over the GFB surface (e.g., Fig. 3*A* Con in sections 312 and 329; Supplemental Videos S2A and S2B). Narrower, shorter first-order efferent vessels (E1) were embedded in mesangium adjacent to the EVC and so had zero podocyte coverage (e.g., Fig. 3*B* E1 in sections 249, 258, and 266). PCB area coverage was estimated in GFB conduit regions (*n* = 10, i.e., Fig. 3; GFB.Con Fig. 4) and small filtration capillary regions (*n* = 22) from four human glomeruli. Conduit vessel PCB area coverage was halved compared with small filtration capillaries (29 ± 3% vs. 55 ± 3%; *P* < 0.0001, *t*-test; Fig. 6*B*).

#### Conduit mesangial support.

Conduit vessels proceed from a central glomerular region with mesangium on all sides (Fig. 4*A* Mes.Con) to regions with less mesangial attachment and areas of filtration barrier (Fig. 4*A* GFB.Con). appendix b shows that moving from mesangial supported regions of conduit to regions where this support is replaced by GFB more than doubles the hoop stress tending to inflate or expand the vessel wall. To test if the GFB conduit regions showed any ballooning due to lack of mesangial support, conduit vessel diameters were measured in 13 glomeruli (resin reconstruction method). Diameters were the same in high (80–100% mesangial cover) and low mesangial cover regions overall (17.7 ± 0.8 µm, 17.9 ± 0.4 µm, respectively, *n* = 61, *P* = 0.28, paired *t*-test). However, after further division of the low mesangial cover data set, conduit vessels with the lowest mesangial cover (<15% mesangium, >85% GFB) showed significant inflation of 7% compared with high mesangial cover regions close to the AVC (*P* = 0.04, paired *t*-test; *P* = 0.04 Wilcoxon; Fig. 6*A*).

### Vascular Resistance and Volume Relationships

Since Poiseuille flow conditions do not apply to an ellipsoidal chamber manifold with many branches, the vascular resistance per unit length could not be calculated for VCs; therefore their capacity, V_AVC_ or V_EVC_, was compared with glomerular vessel resistance parameters. Glomerular volume (V_G_) was used as a correlate of perfusion volume and compared with the resistance parameters.

*R*′_AA_ did not correlate with any of the other *R*′ parameters or V values, and no correlation was found between *R*′_AA_ for afferent arterioles and V_AVC_ (*R*^2^ = 0.014, *P* = 0.68) or V_G_ which it supplies (*R*^2^ = 0.065, *P* = 0.38) (Fig. 2). From the afferent VC there was a significant negative correlation between V_AVC_ and *R*′_Con_ (*R*^2^ = 0.327, *P* = 0.033; Figs. 2 and 6*C*), showing that, as the input manifold gets larger, the supply conduits to the filtration capillary regions get proportionally more conductive (wider).

On the efferent side there was no similar correlation between efferent first-order vessels *R*′_E1_ and V_EVC_ (*R*^2^ = 0.088, *P* = 0.303), although both of these correlated with V_G_, implying a link with perfusion volume. No correlation was found between *R*′_EA_ and V_EVC_ (*R*^2^ = 0.22, *P* = 0.094), but *R*′_EA_ does correlate inversely with V_G_ (*R*^2^ = 0.47, *P* = 0.007; Figs. 2 and 6*D*) and directly with both first-order afferents (*R*′_Con_) and efferents (*R*′_E1_). Figure 2 summarizes the capacity and resistance parameter correlations in the human glomerulus; strikingly, *R*′_AA_ remains independent of all glomerular parameters but all other glomerular vascular entities appear fluid dynamically tied together.

### VC in Single Resin Sections

**Fig. 7.**
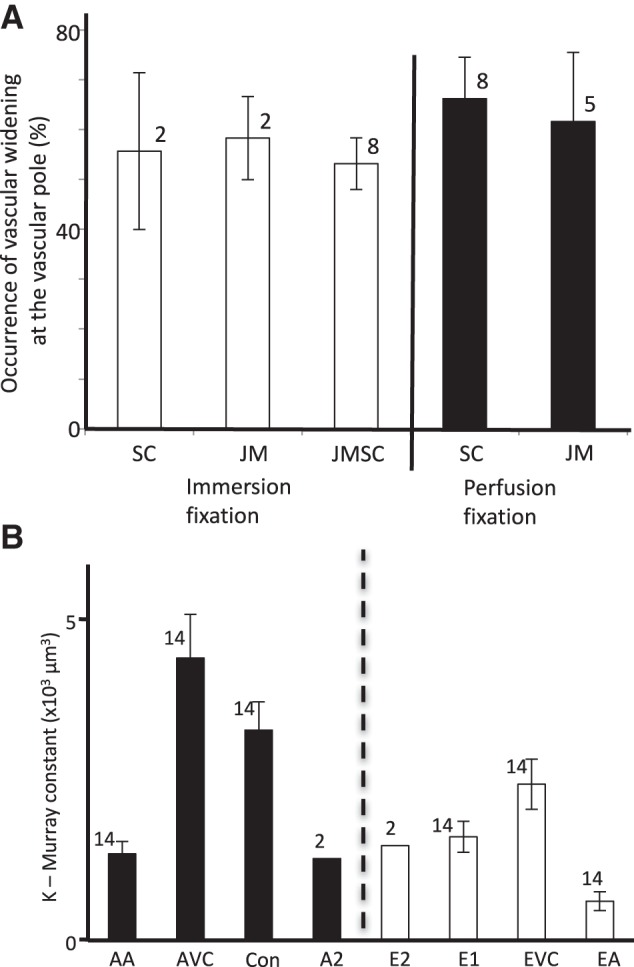
Vascular widenings in single sections. Murray constant from vascular radii. *A*: observed occurrence of glomerular vascular widening in single sections. The frequency with which widening [implying vascular chamber (VC) presence] was observed at vascular poles in immersion and perfusion-fixed glomeruli. SC, subcapsular glomeruli; JM, juxta-medullary glomeruli; JMSC, JM and SC glomeruli combined (*n*, no. of kidneys). *B*: in 14 glomeruli, a Murray constant (*K* = *r*^3^ *n*_V_, where *r* is radius and *n*_V_ is vessel number; see text) was calculated for the afferent and efferent arteriolar tree leading through the VCs and thence into the 1st-order vessels (Con and E1). In 2 glomeruli, *K* was calculated for 2nd-order vessels. The Murray relationship of equal *K* at each vessel level is absent in the afferent VC (AVC), efferent VC (EVC), and conduit (Con) vessels. AA, afferent arteriole; EA, efferent arteriole.

Single sections of immersion- and perfusion-fixed kidney (*n* = 13) revealed randomly orientated profiles of glomeruli with vascular poles (*n* = 177). There was no significant difference in the occurrence of vascular widening at the vascular poles between immersion- and perfusion-fixed glomeruli or between juxta medullary (JM) and subcapsular (SC) glomeruli (Fig. 7*A*). Analysis of all glomeruli together where no discrimination was made in glomerular position (JMSC) in eight immersion-fixed tissues revealed vascular widening in 53 ± 5% of vascular pole sections. Overall frequency was 60 ± 4% for vascular widening in single sections of glomerular vascular poles.

The widened vascular regions found at SC vascular poles were 28.5 ± 3 µm and 30.7 ± 2.1 µm (minimal diameter) after immersion or perfusion fixation, respectively, and represented randomly oriented sections of presumably both vascular chambers. This lack of collapse shows that VCs appear to remain open even when the vascular pressure is reduced during immersion fixation. The full morphology of JM vascular chambers remains to be investigated with serial sections.

### VC Imaged by Confocal and Multiphoton Microscopy

**Fig. 8.**
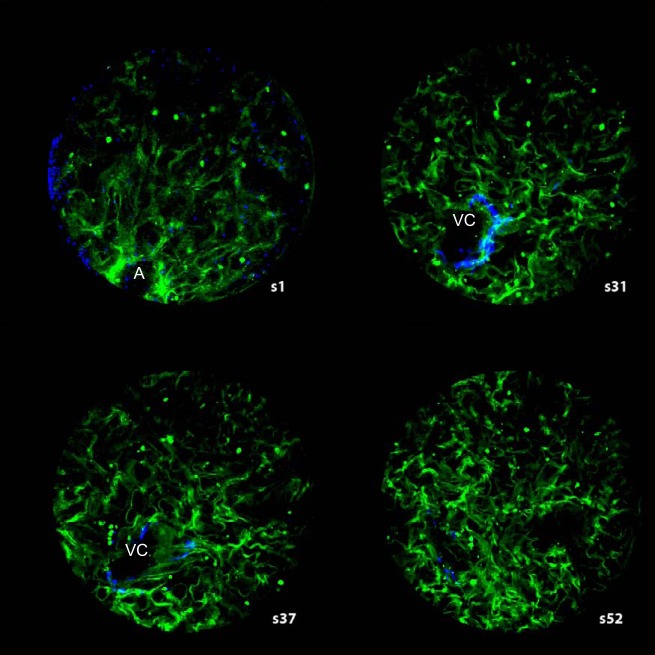
Multiphoton imaging of glomeruli. Images were obtained by combining two-photon fluorescence (TPF) signal images with second harmonic generation (SHG) images of an unfixed human glomerulus. The capillary walls emit a TPF signal (green), with most of the smaller filtration capillaries showing collapse. A banded collagen signal (SHG blue) is located adjacent to a vascular chamber (VC) wall (intense Bowman’s capsule collagen has been blanked). Each optical section 1 µm thick. Section *s1* is close to the tissues’ physical surface; A, arteriole. Section *s31* shows a wide incomplete region of banded collagen around an uncollapsed region (VC) connected with A in *s1*. The banded collagen region has disappeared in section *s37*, but offshoots in attached vessels appear in sections *s37* (*right* of VC) and *s52* (*left* of VC position). Diameter of field, 200 µm. (See Supplemental Video S3 for full section series.)

Using a combination of fixation-induced autofluorescence (FIA), two-photon fluorescence (TPF), and second harmonic generation (SHG) modes, AVC could be seen with attached wide conduit vessels and AA in both fixed and fresh kidney slices (Fig. 8). EVC was more difficult to observe with narrower blood vessels (E1) emerging from them. Measurements of recognized structures show similar dimensions using these optical sectioning methods and resin section reconstruction methods (Table 3).

In addition to morphology, SHG can detect collagen without the need for fixation or labeling. Coherent emission in SHG mode in unfixed glomeruli revealed a signal consistent with banded collagen which when overlaid with coregistered TPF images was positioned in the AVC walls (Fig. 8 and Supplemental Video S3). The collagen sheath extended throughout the AVC and a short distance along the attached conduit vessel walls. A similar banded collagen signal was also seen in fixed tissues. TPF imaging showed fresh glomeruli with extensive vessel collapse in the filtration networks, but VCs appeared resistant to collapse as was found with resin section reconstruction and resin single sections.

### VC Wall Appearance Under Electron Microscopy

**Fig. 9.**
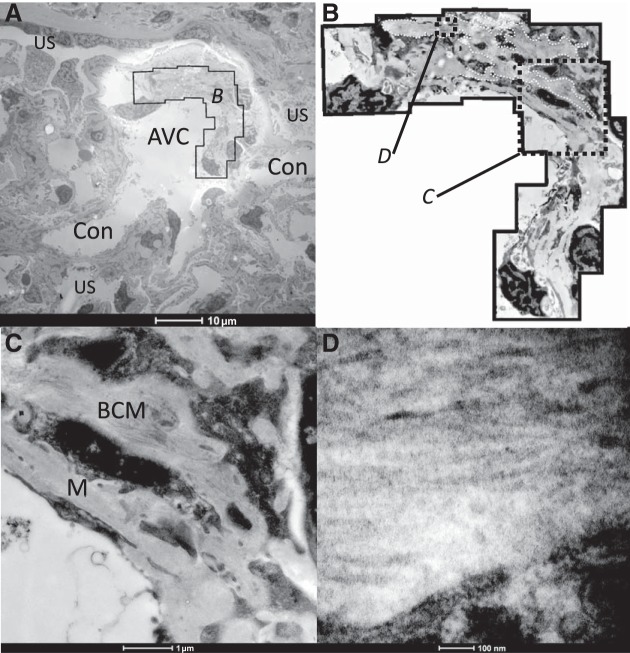
Transmission electron micrographs of vascular chamber walls. Vascular chambers (VC) were imaged using a Tecnai 12 electron microscope. *A*: low power showing vascular pole, an afferent VC (AVC), conduit vessels (Con), and urinary space (US). *B*: montage of micrographs to show the disposition of the banded collagen fibers around the VC walls. White dotted lines show the extent of the mesangial matrix where banded collagen fibers were evident. *C*: area “C” from montage in *B* with matrix rich in banded collagen (BCM) and where collagen is absent (M). *D*: area “D” from montage in *B* with banded collagen fibers.

No visible sign of collagen fibers could be seen in the 1 µm light microscopy resin sections. Electron microscopy sections of AVC showed regions of banded collagen fibers in the surrounding mesangial matrix. The banding was sparse and poorly stained (30 ± 1 nm band spacing), and width of the fibers (30 ± 2 nm) in this partial sheath was consistent with collagen I and III (Fig. 9, *A*–*D*). The collagen bundles extended to a depth of 4 µm from the VC surface (Fig. 9*C* and Table 3). The endothelial lining of AVC contained few fenestrations together with cellular distortions and membrane blebs (Fig. 9*C*), unlike the abundant fenestral density of the filtration capillaries.

## DISCUSSION

### Vascular Chambers

Human glomerular microvascular architecture is not as depicted in current texts. The vascular layout developed over the last 170 years since William Bowman (5) is of a single afferent arteriole which branches until filtration capillaries are reached. These filtration capillaries converge to form a single efferent arteriole conveying blood to the peri-tubular vasculature. This classic picture has been built up from biopsies or necropsies of mammalian kidney.

In human glomeruli both arterioles exhibit vascular widenings more frequently associated with low pressure veins (venous sinuses of the brain) or with large arteries (carotid sinus). However, the glomerular VCs are high-pressure arteriolar afferent and efferent chambers with multiple openings. The closest definition in physical terms is a plenum manifold (plenum: a chamber containing pressurized fluid to control distribution; manifold: a pipe or chamber branching into several openings).

Plenums and manifolds in industry stabilize, distribute, or balance fluid flow through multiple inlets and outlets (i.e., inlet and exhaust manifolds on internal combustion engines). Therefore, our initial hypothesis for glomerular vascular chambers is that they function to balance the pressure and/or flow through the intervening filtration regions without the need for conventional branching within the confined space of the glomerulus. These hemodynamic considerations are not relevant in smaller rodent glomeruli with smaller perfusion volumes relative to arteriolar conductivity (see introduction).

These VC manifolds persist in the glomerulus despite pressure changes. VC walls are resistant to collapse during immersion fixation or when observed fresh at zero pressure. The VC position at the vascular pole allows mesangial structural support and collagen I/III appears to provide (additional) structural integrity. The physiological significance of this collapse resistance is not yet clear.

Collagen III has been observed in glomeruli of collagen nephropathies (7, 14) with collagen III in mesangium and/or capillary walls. No report could be found of collagen I or III in mesangium of normal glomeruli and this report is the first to find banded collagen (I and/or III) in normal glomeruli close to the vascular pole. Banded collagen has previously been found in kidney cortex, where 30 nm fibers showed hybrid labeling with collagen I and III (13); however, the identity of VC wall banded collagen remains to be confirmed by immunohistochemistry.

VCs appear to be ubiquitous in the adult kidney. We confined resin section reconstructions in this study to subcapsular glomeruli to surmount any size difference between subcapsular and juxtaglomerular glomeruli seen in humans and other species (17, 34, 51, 55, 58). Even so, the resin single section work shows a surprisingly similar occurrence of vascular widening in 50–60% of vascular pole glomerular profiles (Fig. 7), implying that VCs exist in both cortical locations with similar sized VCs in both juxta-medullary and subcapsular glomeruli.

### Afferent and Efferent Arterioles

No previous study has measured the diameter of fully opened human glomerular arterioles perfusion fixed at their operating pressures. Previous human AA diameters vary from 13 to 16 µm (18) to diabetic biopsy diameters of 29 µm for AA and 19 µm for EA (44). Other than biological variability, this range of arteriolar diameter is likely due to: volume changes in tissue processing, oblique sections of vessel or low pressure fixation producing collapsed profiles (e.g., Table 3, fresh AA, 13.8 µm; Fig. 1 in Ref. 45). These problems appear minimized with the fixation and resin embedding techniques of this paper.

A correlation between afferent arteriolar diameter and mean glomerular capillary area has previously been seen as consistent with loss of autoregulation (18). Here a correlate of AA resistance per unit length (*R*′_AA_) did not scale with any other glomerular parameter measured including *R*′_EA_ (Fig. 2) preserving the independent autoregulatory control of AA. In contrast, EA resistance per unit length (*R*′_EA_) was inversely correlated with V_G_ (Figs. 2 and 6*D*), and correlating remarkably with R′_Con_ at the afferent end (Fig. 2). Unlike AA, EA is linked in fluid dynamic terms with the Glomerulus it drains.

### Conduit Vessels

The first-order afferent vessels or conduits were noted by Bowman (5) in 1842, with two to eight branches that visibly “subdivide only once or twice as they advance over the surface of the ball.” The few buried deep inside the glomerulus unseen by Bowman may explain the result of 2 to 11 seen in this current study. We also confirm the luminal width of these first-order afferent vessels as being as wide as the efferent arteriole (21).

Conduit vessels show fewer branches than their efferent counterparts, but branch frequency increases at the start of perfusion regions often at some point on the glomerular periphery (Fig. 3). No previous branch data exist for these vessels; however, the interbranch length for all rat glomerular vessels at 26.3 ± 24.9 µm (SD) (48) is between the medians, 32.8 µm (conduit afferent) and 15 µm (efferent) of the skewed distributions found here.

Conduit vessels close to the AVC are embedded in mesangium, and those distal to the AVC have a GFB. While detailed conduit ultrastructure remains to be confirmed, no aberrant GFB capillary morphology has been noted in all of our studies of normal human glomeruli (data not shown). It appears that conduit GFB is similar to filtration capillary GFB except for the scarcity of podocyte cell bodies on the conduit GFB surface. It remains to be determined whether conduit podocytes are just responding to local conditions or are a subpopulation of conduit podocytes with the extralong major processes necessary to cover the GFB area in foot processes.

The GFB is known to remain intact and expand under excess pressure (25, 27), and conduit vessels with a 86–100% GFB—or a sparse 0–14% mesangial attachment around the circumference showed diameter expansion by 7% compared with conduit vessels surrounded by and embedded in mesangium (Fig. 6*A*)—not enough GFB expansion to explain podocyte cell body free areas on the conduit vessels but below the damaged “giant capillary” inflation levels previously reported (25). Conduit inflation might be expected considering the reduced podocyte coverage, thin walls, and wide diameter, and estimates of wall forces show that conduit vessels with a high proportion of GFB and low mesangial attachment are the most susceptible to hoop stress of all glomerular vessels (appendix b). This marks conduits as a target in hypertensive disease, and hoop stress failure has been observed in rat primary afferents (equivalent to conduits) due to glomerular hypertension (with marking albuminuria and glomerulosclerosis) (26).

The subpodocyte space, identified under podocytes (39), should be present under conduit podocyte cell bodies (awaiting electron microscopic confirmation). Incidentally, the light microscopy-derived filtration capillary podocyte cell body (PCB) area coverage of 55% of the GFB fits well with the electron microscopy-derived subpodocyte space coverage of 60% for filtration capillaries found previously (41, 50), suggesting that most of human subpodocyte space is under the podocyte cell body.

### Other Evidence for Vascular Chambers and Conduits

Reconstructed rat glomeruli do not show vascular chambers (48). We confirmed these findings by reconstructing rat glomeruli with Serial Block Face Scanning Electron Microscopy (data not shown) and also found no evidence of VC.

Mammalian arterioles can widen pathologically (32), for instance, mesangiolysis can remove mesangial support causing glomerular vessel aneurysms (35) but such features would not be as highly conserved in shape or have an organized collagenous support as seen in VC found here. Bowman (5) also noted in the larger horse glomerulus that afferent arterioles dilate on the surface before dividing but not in human glomeruli where we have shown that human glomerular vascular dilations are subsurface and would have been invisible to Bowman. The modern conventional description merely reports that the afferent arteriole branches into the glomerular capillary network (22).

VCs may not be present in all human glomeruli. During development, glomerular capillaries arise from one dilated vessel (11), and neonate vascular widening has been shown before the five first-order afferent branches (21) although this has been ascribed to a vessel remnant from the developing nephron (11). Interestingly, the glomerular diameter increase in children from 112 µm (birth) to 167 µm (15 yr) (34) and VC scaling with V_G_ show that VCs may not exist in child glomeruli that are below 160–180 µm diameter, providing that these glomeruli follow the adult glomerular correlation (Fig. 4*B* and Fig. 5, *C* and *D*). Conduit vessel resistance (*R*′_Con_) also scales with V_G_. Whether this correlation continues in smaller (child) glomeruli or whether the primary afferents in children even constitute “conduit” vessels needs evaluation.

Renal biopsies do occasionally show evidence of VCs and conduit vessels in section, and a survey of images in biomedical journals reveals light micrographs showing a 15 µm conduit vessel and 20 µm VC (20), a 30 µm diameter VC (52), and VCs at both efferent and afferent ends (44). However, without the context of a serial section stack, these micrographs remain as widened vascular profiles.

VCs could be artefacts of processing volume changes; however, glomerular diameters (~200 µm) derived here were between immersion-fixed (160–170 µm) (10, 31) and autopsy diameters (260–270 µm) (8) and closely match in vivo ultrasound values of 200 µm (15, 23), suggesting that glomerular volume changes during processing were minimal overall.

Wide profiles at the vascular pole in singles sections can be dismissed as collapsed vessels. Put simply, an afferent arteriole terminus of 21 µm diameter with a circumference of 66 µm could conceivably collapse to a flattened squashed-circle profile ~30 µm wide that, if sectioned longitudinally, would fit exactly with the 28–30 µm wide profiles measured; however, 60% of randomly oriented single sections of vascular poles all showed these wide vascular regions—far too frequent for the collapse argument. Additionally, in this study, vessel collapse was seen in filtration capillaries in fresh glomeruli (multiphoton microscope: Supplemental Video S3) but with VCs held open. VCs are not collapse artefacts but stiff walled vascular structures.

### The Murray Relationship

The relationship between branching vessel diameters was derived by Murray on the principle of minimum work for blood flow (36, 37) where the radius cubed of the parent vessel equals the sum of the cubes of the daughter vessel radii. The Murray relationship holds for arteries and venules of rat kidney down to the afferent arterioles and venules leading away from the tubular networks (42), but it is not known if it continues into the glomerulus. The Murray relationship in whole human kidney also remains to be assessed.

A Murray constant (*K*) was calculated for each set of vessels leading into and away from human glomerular VCs in all 14 glomeruli reconstructed from resin sections:

(2) $$K=r^{3}n_{\text{V}}$$

where *n*_V_ is the number of vessels and *r* is the radius. Using *r*_AA_, *r*_AVC_, *r*_Con_, *r*_E1_, *r*_EVC_, *r*_EA_, and appropriate *n* to calculate *K*, the Murray relationship breaks at the VCs and the first-order vessels (conduit and E1 vessels; Fig. 7*B*), where daughter vessels do not have the same Murray constant as parent vessels.

This is an exception to Murray’s law, a plenum/manifold exception, where flow distribution from a single arteriole provides a high-pressure distributive flow into many glomerular lobes in a short distance. An estimate of *K* values for second-order afferent vessels (A2 in 2 glomeruli) showed that *K* may return to the value predicted by the afferent arteriolar radius after skipping the VC and conduit vessels (Fig. 7*B*). Other Murray’s law exceptions occur where a higher surface area is required in the exchange vessels of an organ, for instance alveolar capillary networks (59).

The possible mechanisms producing a set of vessels following Murray’s law includes an endothelial transducer triggering remodeling after a shear force threshold was exceeded (46). Altering the threshold could induce the vessel diameter changes seen here. However, the Murray relationship requires laminar flow through vessels and the hemodynamic flow will be complex from an afferent arteriole into an ellipsoidal vascular chamber with several outlets.

### VC Hemodynamics

If glomerular volume is used as a measure of perfusion capacity, it rises and falls along with the size of the AVC and the EVC (Fig. 5, *C* and *D*). Larger AVCs feed more blood to larger glomerular filtration regions and thence to larger EVCs. As the size increases, the resistance of the conduit vessels, E1 and EA (not AA), falls to accommodate the flow (vessels get wider in proportion to Poiseuille flow) (Fig. 6, *C* and *D*). All of the major vessels of the human glomerulus past the afferent arteriole are linked in some way in terms of flow and capacity (Fig. 2). How would flow progress from laminar flow in an afferent arteriole through the AVC to the conduit vessels? And similarly from efferent E1 vessels through EVC to the efferent arterioles?

A clue to VC flow characteristics comes from the kinks and bends in AAs. One constant feature of the glomeruli analyzed is the bend as the afferent arteriole enters the AVC. These bends can be readily seen in the glomeruli of Fig. 1 and Fig. 3, *A* and *B* (Supplemental Videos S2A and S2B) and showed an average 60° deviation from a straight path. The fluid flow at a bend in a channel is known to induce vortices (49), and we hypothesize that the summation of all bends in an afferent arteriole (i.e., see bend from interlobular–AA junction in Fig. 1) could induce a single major vortex in the AVC, possibly aiding distributive flow centrifugally into conduit vessels.

If such a vortex with its axis in the midline of the AVC adopts the properties of a “rigid-body” or “rotational” vortex, then the pressure at the AVC edge at the conduit vessel openings would depend both on the hydrostatic pressure and on the dynamic pressure (set by the angular momentum of the moving fluid – ½ρω^2,^ where ρ is  density and ω is angular velocity). Crucially, however, the dynamic pressures within this form of vortex are uniform (3).

We speculate that in health, the AVC and the complex (vortical) fluid movement within it may ensure a uniform driving pressure into the conduit vessels, maximizing a uniform distribution of flow to each of the glomerular lobules. The loss of this equalizing distributary mechanism, through microvascular disease, mesangial proliferation occluding the AVC, hyperperfusion or immunological injury, could potentially result in localized hyperfiltration and excess shear stress in some glomerular segments with stasis in others. This has implications for glomerular disease in which only some perfused regions of the glomerulus appear to have sustained sclerotic/fibrotic damage [e.g., focal segmental glomerulosclerosis (FSGS)] whereas adjacent lobules appear normal.

The structure of the efferent vascular chamber, with many microvessels converging on a chamber, lends itself to the development of an irrotational vortex (plug hole vortex) balancing EVC pressure gradients and promoting balanced removal of blood from the glomerular tuft (3).

### Conclusion

We show for the first time in human glomeruli that clearly defined afferent arterioles lead into afferent vascular chambers of ellipsoid shape and structure embedded in the mesangium of the glomerular vascular pole and ensheathed in collagen fibrils. These chambers are plenum manifolds with many emergent relatively unbranched wide blood vessels or conduits conveying blood to the periphery of the glomerulus. Branching frequency increases at the end of the conduits, leading to filtration capillary networks that lead back to smaller efferent vascular chambers in the mesangium of the vascular pole and then the efferent arteriole. The conduit vessels are sparsely covered with podocytes, and conduit fluid resistance scales with the size of the afferent vascular chambers. Both vascular chambers scale with glomerular capacity, suggesting absence of vascular chambers in glomeruli below 160 µm diameter (the glomeruli of children). Resistance correlates of first-order afferent (conduit) and efferent vessels and efferent arterioles (but not afferent arterioles) scale together and inversely with glomerular volume. We propose that all of these structures represent a large glomerulus adaptation allowing even hemodynamic flow distribution and pressure balance across the many lobes of a human glomerulus.

## GRANTS

This study was financed by The Richard Bright Research Trust until 2014 and then by Kidney Research UK (2014-2015), Medical Research Council Grants G10002073 (S. J. Harper) and G0802829 (A. H. J. Salmon).

## DISCLOSURES

No conflicts of interest, financial or otherwise, are declared by the authors.

## AUTHOR CONTRIBUTIONS

C.R.N. conceived and designed research; C.R.N., K.P.A., J.S.B., and K.B.B. performed experiments; C.R.N., K.P.A., J.S.B., K.B.B., and S.J.H. analyzed data; C.R.N., K.P.A., J.S.B., D.O.B., C.P.W., A.H.J.S., and S.J.H. interpreted results of experiments; C.R.N. prepared figures; C.R.N. drafted manuscript; C.R.N., K.P.A., J.S.B., D.O.B., C.P.W., A.H.J.S., and S.J.H. edited and revised manuscript; C.R.N. and S.J.H. approved final version of manuscript.

## Footnotes

1 Afferent arteriole conductance estimated from the 4th power of vessel radii [mice, *r* = 5–6.5 µm (16, 28, 29); rats, *r* = 7–9.5 µm (12, 24, 54, 57); human, *r* = 11 µm (this article)], with human glomerular volume estimated from glomerular diameter (mouse = 70 µm, rat = 120 µm, and human = 200 µm).

## APPENDIX A

### Vascular Resistance

The vascular resistance to flow will change as blood flows along AA into AVCs and conduits and later pools in EVCs before flowing into EA. To better understand how blood flow is affected by the changing morphology, a correlate of vascular resistance *R*′_Con_) was derived from the Poiseuille equation using vessel radii and vessel number. For VCs the flow will be complex and nonlaminar in the spheroidal shape and so the Poiseuille equation could not be used so VC volume was used as a measure of VC capacity.

### Resistance Changes in Arterioles and Conduit Vessels

For conduit vessel resistance (∑*R*_Con_) coming out of the afferent VC where *R*_Con3_ is the resistance of the 3rd conduit vessel in parallel:

(A1.1) $$\frac{1}{\sum R_{\text{Con}}}=\frac{1}{R_{\text{Con1}}}+\frac{1}{R_{\text{Con2}}}+\frac{1}{R_{\text{Con3}}}. . .\frac{1}{R_{\text{Con}n}}$$

For *n*_Con_ similar conduit vessel resistances *R*_ConX_

(A1.2) $$\frac{1}{\sum R_{\text{Con}}}=\frac{n_{\text{Con}}}{R_{\text{Con}X}}$$

For fluid of viscosity η, the resistance to flow through a tube of length *L* is inversely proportional to the 4th power of the radius (Poiseuille’s law), similarly:

(A1.3) $$R_{\text{Con}X}=\frac{8\eta_{\text{Con}}L_{\text{Con}}}{\pi r_{\text{Con}}{}^{4}}$$

where *L*_Con_ is conduit vessel length and *r*_Con_ is the mean conduit vessel radius. If the viscosity of the blood flowing through VC and attached vessels (η_Con_) is assumed not to change (low filtration into mesangium in these vessels) then η_Con_ with π and 8 can be combined into a constant *k*_Con_:

(A1.4) $$R_{\text{Con}X}=\frac{k_{\text{Con}}L_{\text{Con}}}{r_{\text{Con}}{}^{4}}$$

Combining *Eqs. A1.2* and *A1.4*:

(A1.5) $$\frac{1}{\sum R_{\text{Con}}}=\frac{r_{\text{Con}}{}^{4}n_{\text{Con}}}{k_{\text{Con}}L_{\text{Con}}}$$

Inverting *Eq. A1.5* and dividing by *L*_Con_ and *k*_Con_ yields a measure of the total conduit vessel resistance per unit length (*R*′_Con_).

(A1.6) $$\frac{\sum R_{\text{Con}}}{L_{\text{Con}}k_{\text{Con}}}=\frac{1}{r_{\text{Con}}{}^{4}n_{\text{Con}}}={R^{\prime }}_{\text{Con}}$$

1/*r*_Con_^4^ *n*_Con_ was used to estimate a correlate of vascular resistance per unit length of all conduit vessels in parallel (*R*′_Con_). Similarly, 1st-order efferents were assessed using 1/*r*_E1_^4^ *n*_E1_. (*R*′_E1_). Correlates of afferent and efferent arteriole resistance per unit length (*R*′_AA_, *R*′_EA_) were estimated with 1/*r*_AA_^4^ and 1/*r*_EA_^4^.

## APPENDIX B

### Vascular Wall Stress

The conduit vessel wall morphology appears similar to filtration capillaries however, conduits are much wider. Greater-diameter tubes or vessels of the same wall thickness are more susceptible to pressure damage or rupture. How might conduit vessel wall stress compare with other glomerular vessels?

### VC and Conduit Vessel Wall Stress

The effective wall strength and compliance of systemic capillaries are largely due to basement membrane/basal lamina (40). Assuming that glomerular vascular wall strength is due to the glomerular basement membrane (GBM, 0.3 µm and less than 1/10th of vessel radius) then the Laplace equation (60) can be used to derive the hoop stress (*S*_h_) of the vascular wall (the force exerted circumferentially trying to pull the wall apart). For cylindrical conduit vessels:

(A2.1) $$S_{\text{hCon}}=\frac{\Delta P_{\text{Con}}r_{\text{Con}}}{t_{\text{Con}}}$$

where *∆P*_Con_ is the hydrostatic pressure difference across conduit vessel wall of radius *r*_Con_ and effective wall thickness *t*_Con_.

The equation for a near spherical VC is half that of an equivalent diameter cylinder:

(A2.2) $$S_{\text{hVC}}=\frac{\Delta P_{\text{VC}}r_{\text{VC}}}{2t_{\text{VC}}}$$

where *∆P*_VC_ is the hydrostatic pressure difference across the VC wall of radius *r*_VC_ and effective wall thickness *t*_VC_ . The effective strength of the arteriolar wall will be a composite of strengths of this thick multilayered structure; however, the arteriole smooth muscle wall thins as it transitions into the VC, with only endothelium, basal lamina, and collagen sheath surrounded by mesangial matrix.

### Hoop Stress Calculations

#### Vascular hoop stress for AVC.

Parameters were as follows: *r*_AVC_ *=* 22 µm (mean of *r*′_AVC_, *r*′′_AVC_, *r*′′′_AVC_; Table 1); *t*_AVC_ *=* 0.5–4 µm [between the first mesangial lamina thickness ~0.5 µm (see Fig. 9*D*) and the collagen sheath dispersed over 4 µm (Table 3 and Fig. 9); *∆P*_AVC_ = 23 mmHg [AVC luminal pressure of 63 mmHg (43) minus mesangial pressure—a high proportion of capillary hydrostatic pressure (9)— likely 40 mmHg because mesangial cells respond to 40 mmHg and above (19, 30).

*S*_hAVC_ = 8–66 kPa (*Eq. A2*.*2*) [≤8 kPa if the effective ∆*P*_AVC_ is lower due to pressure dissipating gradients and effective *t*_AVC_ is thicker due to additional mesangial matrix support (7)].

#### Vascular hoop stress for mesangial conduit vessels.

The mesangial backed conduit (MC) vessels (Fig. 4*A*, Mes.Con) adjacent to AVCs would share the same mesangial protection and possibly collagen sheath as the AVCs: *r*_MC_ = 8 µm (Table 1); *t*_MC_ = 0.5 µm to 4 µm (Table 3 and Fig. 9); ∆*P*_MC_ = 23 mmHg (see above).

*S*_hMC_ = 6–48 kPa (*Eq. A2*.*1*) (≤6 kPa, *S*_hAVC_ caveat as above).

#### Vascular hoop stress for GFB conduit vessels.

The conduit vessels away from the AVC are connected to mesangium only on a small part of their circumference, the rest being normal GFB and GBM (Fig. 3 and Fig. 4*A* GFB Con): *r*_GC_ = 8 µm (GC, GFB conduit wall; Table 1); *t*_GC_ = 0.3 µm (GBM thickness); ∆*P*_GC_ = 38 mmHg [luminal *P* (63 mmHg) minus urinary space *P* (25 mmHg)].

*S*_hGC_ = 133 kPa (*Eq. A2*.*1*).

#### Vascular hoop stress for filtration capillaries.

Parameters are as follows: *r*_FC_ = 3.5 µm; *t*_FC_ = 0.3 µm (GBM thickness; FC, filtration capillary); ∆*P*_FC_ = 38 mmHg [luminal *P* (63 mmHg) minus urinary space *P* (25 mmHg)].

*S*_hFC_ = 58 kPa (*Eq. A2*.*1*).

Hoop stress (*S*_h_) calculations are shown in Table A1. *S*_h_ is difficult to estimate in the mesangial backed AVC and mesangial conduit vessels, but our maximum estimate is less than half the value for the GFB conduit. *S*_h_ falls in the filtration capillaries of the same wall thickness, but these are protected by their small radius. At the efferent end the reduced radii and complete mesangial encasement of VC and of the short E1 vessels would result in lower *S*_h_ of the equivalent efferent vessels (not shown).

In conclusion, in human glomeruli, GFB conduit walls (GC) mark a peak of hoop stress caused by the relatively thin wall for the large diameter. While the AVC and the early conduit vessel are protected by mesangial backing, any mesangial disruption through immune-mediated damage, cell invasion or proliferation or disruption to the collagen sheath will change S_hAVC_ and S_hMC_ making AVC and mesangial conduit vessels vulnerable to pressure changes.

## Appendix

**Table A1.** Calculated vascular hoop stress *S*_h_

	Subscript Abbreviation	Δ*P*, mmHg	*r*, µm	*t*, µm	*S*_h_, kPa
Afferent VC	AVC	23	22	0.5–4.0	≤8–66
Mesangial conduit	MC	23	8	0.5–4.0	≤6–48
GFB conduit	GC	38	8	0.3	133*
Filtration capillary	FC	38	3.5	0.3	58

* The peak is in the glomerular filtration barrier (GFB) conduit vessels.
